# Protein Biomarkers and Major Cardiovascular Events in Older People With Advanced CKD: The European Quality (EQUAL) Study

**DOI:** 10.1016/j.xkme.2023.100745

**Published:** 2023-11-02

**Authors:** Samantha J.L. Hayward, Nicholas C. Chesnaye, Barnaby Hole, Ryan Aylward, Yvette Meuleman, Claudia Torino, Gaetana Porto, Maciej Szymczak, Christiane Drechsler, Friedo W. Dekker, Marie Evans, Kitty J. Jager, Christoph Wanner, Fergus J. Caskey, EQUAL investigators

**Affiliations:** 1 Translational Health Sciences, Bristol Medical School, University of Bristol, Bristol, United Kingdom; 2 Southmead Hospital, North Bristol NHS Trust, Bristol, United Kingdom; 3 Amsterdam UMC, University of Amsterdam, ERA Registry, Medical Informatics, Amsterdam, The Netherlands; 4 Amsterdam Public Health Research Institute, Quality of Care, Amsterdam, The Netherlands; 5 Population Health Sciences, Bristol Medical School, University of Bristol, Bristol, United Kingdom; 6 Department of Medicine, Faculty of Health Sciences, University of Cape Town, South Africa; 7 Department of Clinical Epidemiology, Leiden University Medical Center, Leiden, The Netherlands; 8 Institute of Clinical Physiology, National Research Council, Reggio Calabria, Italy; 9 GOM Bianchi Melacrino Morelli, Reggio Calabria, Italy; 10 Department of Nephrology and Transplantation Medicine, Wroclaw Medical University, Wroclaw, Poland; 11 Division of Nephrology, University Hospital of Wurzburg, Wurzburg, Germany; 12 Department of Clinical Sciences Intervention and Technology, Karolinska Institutet and Karolinska, Stockholm, Sweden

**Keywords:** Biomarker, cardiovascular disease, chronic kidney disease

## Abstract

**Rationale & Objective:**

Cardiovascular disease is the leading cause of morbidity and mortality in chronic kidney disease (CKD). We investigated 184 inflammatory and cardiovascular proteins to determine their potential as biomarkers for major cardiovascular events (MACEs).

**Study Design:**

The European Quality (EQUAL) is an observational cohort study that enrolled people aged ≥65 years with an estimated glomerular filtration rate ≤20 mL/min/1.73 m^2^.

**Setting & Participants:**

Recruited participants were split into the discovery (n = 611) and replication cohorts (n = 292).

**Exposure:**

Levels of 184 blood proteins were measured at the baseline visit, and each protein was analyzed individually.

**Outcome:**

MACE.

**Analytical Approach:**

Cox proportional hazard models adjusted for age, sex, estimated glomerular filtration rate, previous MACE, and country were used to determine the risk of MACE. Proteins with false discovery rate adjusted *P* values of <0.05 in the discovery cohort were tested in the replication cohort. Sensitivity analyses were performed by adjusting for traditional risk factors, CKD-specific risk factors, and level of proteinuria and segregating atherosclerotic and nonatherosclerotic MACE.

**Results:**

During a median follow-up of 2.9 years, 349 people (39%) experienced a MACE. Forty-eight proteins were associated with MACE in the discovery cohort; 9 of these were reproduced in the replication cohort. Three of these proteins maintained a strong association with MACE after adjustment for traditional and CKD-specific risk factors and proteinuria. Tenascin (TNC), fibroblast growth factor-23 (FGF-23), and V-set and immunoglobulin domain-containing protein 2 (VSIG2) were associated with both atherosclerotic and nonatherosclerotic MACE. All replicated proteins except carbonic anhydrase 1 and carbonic anhydrase 3 were associated with nonatherosclerotic MACE.

**Limitations:**

Single protein concentration measurements and limited follow-up time.

**Conclusions:**

Our findings corroborate previously reported relationships between FGF-23, vascular cell adhesion protein-1, TNC, and placental growth factor with cardiovascular outcomes in CKD. We identify 5 proteins not previously linked with MACE in CKD that may be targets for future therapies.

**Plain-Language Summary:**

Kidney disease increases the risk of heart disease, stroke, and other vascular conditions. Blood tests that predict the likelihood of these problems may help to guide treatment, but studies are needed in people with kidney disease. We analyzed blood tests from older people with kidney disease, looking for proteins associated with higher risk of these conditions. Nine proteins were identified, of which 3 showed a strong effect after all other information was considered. This work supports previous research regarding 4 of these proteins and identifies 5 additional proteins that may be associated with higher risk. Further work is needed to confirm our findings and to determine whether these proteins can be used to guide treatment.

People with chronic kidney disease (CKD) are at high risk of cardiovascular disease, and this risk increases both with age and CKD progression.^1^^,^^2^ Indeed, major adverse cardiovascular events (MACEs) are the leading cause of death for people with CKD, accounting for approximately a third of deaths.^3^ Although traditional risk factors remain relevant in people with CKD, additional CKD-specific risk factors, such as anemia, calcium and phosphate dysregulation, uremic toxins and chronic inflammation may also contribute to the high risk of MACE.^4^

Biomarkers for CKD-associated MACE could guide prognostication, inform design of stratified clinical trials, and highlight proteins involved in novel CKD-specific MACE mechanisms. However, the identification of biomarkers in advanced CKD is complicated by decreased renal clearance affecting plasma protein concentrations. For example, although brain natriuretic peptide, troponin, and C-reactive protein are useful cardiovascular biomarkers in the general population, all 3 can be persistently increased in people with CKD, changing their relationship with outcomes in such populations.^5^^,^^6^ The discovery of clinically translatable biomarker results necessitates specific MACE biomarker studies in people with advanced CKD.

Older people living with advanced CKD are often excluded from biomarker studies or comprise too small a proportion of the study cohort to elicit results that are generalizable to this subgroup. This is an important weakness of the current literature given the especially high risk of cardiovascular disease experienced by older people with CKD.^7^ We investigated the association between 184 cardiovascular and inflammatory proteins and the risk of MACE in a cohort of older people with advanced CKD. In addition, we explored whether the relationship between these proteins and MACE risk was attenuated by traditional risk factors, CKD-specific risk factors, or level of proteinuria and whether these proteins were associated with atherosclerotic or nonatherosclerotic MACE.

## Methods

### Study Cohort

The European Quality (EQUAL) Study is a prospective cohort study of older people with advanced CKD recruited from 6 countries. Inclusion criteria were age ≥65 years and an incident estimated glomerular filtration rate (eGFR) of ≤20 mL/min/1.73m^2^.^8^ Participants were excluded if the decrease in eGFR was the result of an acute event or if they had received kidney replacement therapy before study recruitment.^9^ Approval was obtained from the medical ethical committees or institutional review boards for all participating countries, and written informed consent was obtained from all participants in adherence to the Declaration of Helsinki.

Nine hundred twenty-one of the EQUAL participants had serum samples available. The recruits were split a priori into a discovery cohort (n = 618, recruited from the United Kingdom, Germany, and Poland) and a replication cohort (n = 303, recruited from Sweden).

### Clinical Data

Demographic and clinical data were collected by research nurses using a case report form completed in person and corroborated against the patient’s medical notes. Follow-up clinical data were collected in the same manner at 3-6 monthly intervals. eGFR was calculated using the 2009 Chronic Kidney Disease Epidemiology Collaboration (CKD-EPI) equation.^10^ For patients who lacked urinary albumin-creatinine ratio (uACR) data, uACR values were estimated from urinary protein-creatinine ratios using the equation described by Weaver et al.^11^

### Protein Data

Peripheral blood samples were collected at the participants’ first study visit. The samples were collected following standardized operating procedures and were stored at −80 °C. The samples were tested for 184 different proteins using the Olink Target 96 Cardiovascular II and Cardiometabolic protein panels (www.Olink.com; full protein list including abbreviations are available in Table S1). The high throughput Olink panels detect protein abundance by proximity extension assay technology; oligonucleotide-labelled antibody probe pairs bind to the target protein and are amplified by polymerase chain reaction and quantified.^12^ The protein data were transformed on a log2 scale for analysis; therefore, all the reported hazard ratios relate to a doubling of protein level.

Quality control of the protein data was performed using 3 intrasample controls and 8 plate controls. The internal controls were added to every sample to assess each step of the proximity extension assay process; 12 samples were excluded from the Cardiovascular II panel and 3 from the Cardiometabolic panel as the internal controls failed to reach the expected values. In total, 11 proteins failed to reach the limit of detection calculated from the plate controls in >50% of the samples and were removed from further analyses (Table S1). After quality control was performed, data were available for 173 proteins for 611 participants in the discovery cohort and 292 participants in the replication cohort.

### Outcome Definition

A variety of MACE definitions are used in observational research.^13^ For the primary analysis, a purposefully broad composite MACE definition was used as follows: the first episode during follow-up of cerebrovascular disease, myocardial infarction (MI), angina, congestive heart failure (CHF), coronary artery disease, arrhythmia, or peripheral vascular disease (PVD) or death from cerebrovascular accident, MI, CHF, or cardiac arrest during study follow-up. More specific MACE definitions were explored in the sensitivity analyses (see below).

### Statistical Analysis

Univariable and multivariable Cox proportional hazards models with mixed effects were used to investigate the risk of MACE for each individual protein in a complete case analysis. In the primary analysis, the multivariable models were adjusted for the potential confounders of age, sex, eGFR, previous MACE (fixed effects) and country (random effect). The random effect of country was included for the discovery cohort only, as this cohort included participants from multiple countries. *P* values were adjusted for multiple comparisons using the Benjamini and Hochberg method (false discovery rate [FDR]).^14^ Only proteins with FDR-adjusted *P* values < 0.05 in the discovery cohort models proceeded to testing in the replication cohort. Functional annotation and enrichment analysis were performed using the Protein Annotation Through Evolutionary Relationship Pathways annotation data set (pantherdb.org) and g:Profiler (biit.cs.ut.ee/gprofiler).^15^^,^^16^ As only proteins with a role in inflammation or cardiovascular disease were studied, the g:Profiler scope selection domain was set to the 173 protein list rather than all known proteins to prevent selection bias from influencing the results of the enrichment analysis. Protein-protein interactions were determined using the Search Tool for the Retrieval of Interacting Genes/Proteins data set (STRING; string-db.org).^17^ R version 4.1.2 was used for analysis.

### Sensitivity Analyses

Additional multivariable Cox proportional hazard analyses were performed on any replicated protein using discovery cohort data only. The first sensitivity analyses explored whether the association between the protein and risk of MACE was independent of traditional cardiovascular risk factors, CKD-specific risk factors, and level of proteinuria. Traditional risk factors were defined as history of hypertension or diabetes, smoking status, blood pressure, body mass index and cholesterol at first study visit; data were available for 275 participants. CKD-specific risk factors were defined as hemoglobin, calcium, phosphate, and parathyroid hormone concentrations at first study visit; data were available for 408 participants. The proteinuria sensitivity analysis adjusted for uACR at the first study visit, and data were available for 239 recruits.

In the second sensitivity analyses, the MACE outcome was split into atherosclerotic MACE (defined as cerebrovascular disease, MI, angina, coronary artery disease or PVD or death from cerebrovascular accident or MI) and nonatherosclerotic MACE (defined as CHF, arrhythmia, or death from CHF or cardiac arrest). If the cardiovascular event consisted of concurrent pathologies (ie, MI and CHF) or if any of the pathologies met the atherosclerotic definition, the event was classed as an atherosclerotic MACE. All the sensitivity analyses were also adjusted for age, sex, eGFR, previous MACE (fixed effects) and country (random effect).

## Results

The baseline demographic and clinical characteristics of the discovery and replication cohorts are listed in Table 1. There were a smaller proportion of males and a greater proportion of kidney failure of uncertain cause in the discovery cohort compared with the replication cohort. More patients in the replication cohort had hypertension and kidney failure due to hypertensive nephropathy. All other clinical and demographic characteristics were broadly similar across the 2 cohorts.

**Table 1:** Baseline Demographic and Clinical Characteristics of the Patient Cohorts

Characteristic		Discovery Cohort N = 611	Replication Cohort N = 292
Age (y)		77 (71, 82)	76 (70, 80)
Sex	Male	374 (61%)	205 (70%)
Ethnicity	White	573 (94%)	287 (98%)
	Missing data	3 (<1%)	1 (<1%)
eGFR (mL/min/1.73 m^2^)		18 (14, 21)	18 (15, 20)
Primary renal diagnosis	Hypertension	175 (29%)	127 (43%)
	Diabetes	127 (21%)	63 (22%)
	Kidney failure of uncertain cause	119 (19%)	12 (4%)
	Glomerular	58 (9%)	32 (11%)
	Tubulointerstitial	62 (10%)	25 (9%)
	Missing data	3 (<1%)	2 (<1%)
Comorbid conditions	Diabetes	256 (42%)	111 (38%)
	Missing data	10 (2%)	1 (<1%)
	Hypertension	496 (81%)	267 (91%)
	Missing data	31 (5%)	1 (<1%)
	Myocardial infarction	89 (15%)	51 (17%)
	Missing data	9 (1%)	1 (<1%)
	Cerebrovascular disease	81 (13%)	51 (17%)
	Missing data	16 (3%)	1 (<1%)
	Peripheral vascular disease	86 (14%)	35 (12%)
	Missing data	25 (4%)	1 (<1%)
Medications	Lipid modifying agents	409 (67%)	160 (55%)
	Beta blockers	334 (55%)	179 (61%)
	Renin-angiotensin inhibitors	279 (46%)	191 (65%)
Charlson comorbidity index		7 (6, 8)	7 (6, 8)
	Missing data	10 (2%)	1 (<1%)
Body mass index (kg/m^2^)		29 (25, 33)	27 (24, 30)
	Missing data	53 (9%)	1 (<1%)
Smoking status	Ex-smoker	249 (41%)	153 (53%)
	Nonsmoker	190 (31%)	111 (38%)
	Smoker	36 (6%)	22 (8%)
	Missing data	136 (22%)	6 (2%)
Blood pressure (mm/Hg)	Systolic	144 (130, 160)	148 (131, 160)
	Diastolic	73 (66, 80)	76 (67, 85)
	Missing data	6 (1%)	0
Hemoglobin (mmol/L)		7.07 (6.45, 7.82)	7.32 (6.75, 7.88)
	Missing data	16 (3%)	0
Cholesterol (mmol/L)		4.40 (3.60, 5.31)	4.60 (3.70, 5.50)
	Missing data	213 (35%)	8 (3%)
Calcium (mmol/L)		2.29 (2.20, 2.38)	2.28 (2.19, 2.37)
	Missing data	58 (9%)	3 (1%)
Phosphate (mmol/L)		1.26 (1.11, 1.44)	1.30 (1.10, 1.50)
	Missing data	46 (8%)	0
Parathyroid hormone (pmol/L)		16 (9, 25)	15 (10, 22)
	Missing data	164 (27%)	9 (3%)
Urine ACR (mg/mmol)		45 (6, 174)	39 (10, 169)
	Missing data	375 (61%)	45 (15%)

*Note:* N (%), median (interquartile range).

Abbreviations: ACR, albumin:creatinine ratio; eGFR, estimated glomerular filtration rate.

### MACE Outcomes: Discovery and Replication Cohorts

After a median follow-up of 2.9 years, 349 (39%) recruits had experienced MACE, and 283 (31%) people had died. Eighty-eight (31%) of these deaths were attributed to MACE. There were marginally fewer MACE in the discovery cohort; however, the median follow-up time was slightly shorter (2.7 years compared with 3.5 years). The proportional contribution of each type of clinical event to the MACE outcome differed between the 2 cohorts, with more PVD observed in the discovery cohort and more arrhythmias, CHF, and nonatherosclerotic MACE deaths in the replication cohort. A breakdown of the nature of first MACE for each cohort is provided in Table 2.

**Table 2:** Clinical Events Contributing to MACE Outcomes

First MACE Breakdown	Discovery Cohort N = 611	Replication Cohort N = 292
**First MACE - all**	**219 (36%)**	**130 (45%)**
Atherosclerotic MACE	135 (22%)	63 (22%)
Myocardial infarction	22 (4%)	10 (3%)
Angina	7 (1%)	6 (2%)
Coronary artery disease	17 (3%)	1 (<1%)
Cerebrovascular event	24 (4%)	14 (5%)
Peripheral vascular disease	47 (8%)	10 (3%)
Multiple simultaneous diagnoses including an atherosclerotic MACE	4 (<1%)	12 (4%)
Atherosclerotic MACE death∗	14 (2%)	10 (3%)
**Nonatherosclerotic MACE**	84 (14%)	67 (23%)
Heart failure	40 (7%)	21 (7%)
Arrhythmia	30 (5%)	25 (9%)
Multiple simultaneous non-atherosclerotic MACE diagnoses	1 (<1%)	6 (2%)
Nonatherosclerotic MACE death	13 (2%)	15 (5%)

*Note:* N (%). This table lists a breakdown of first MACE. In total, 88 patients died of MACE; however, 36 of these had another MACE before their death.

### Univariable and Multivariable Analyses: Discovery Cohort

**Figure 1:**
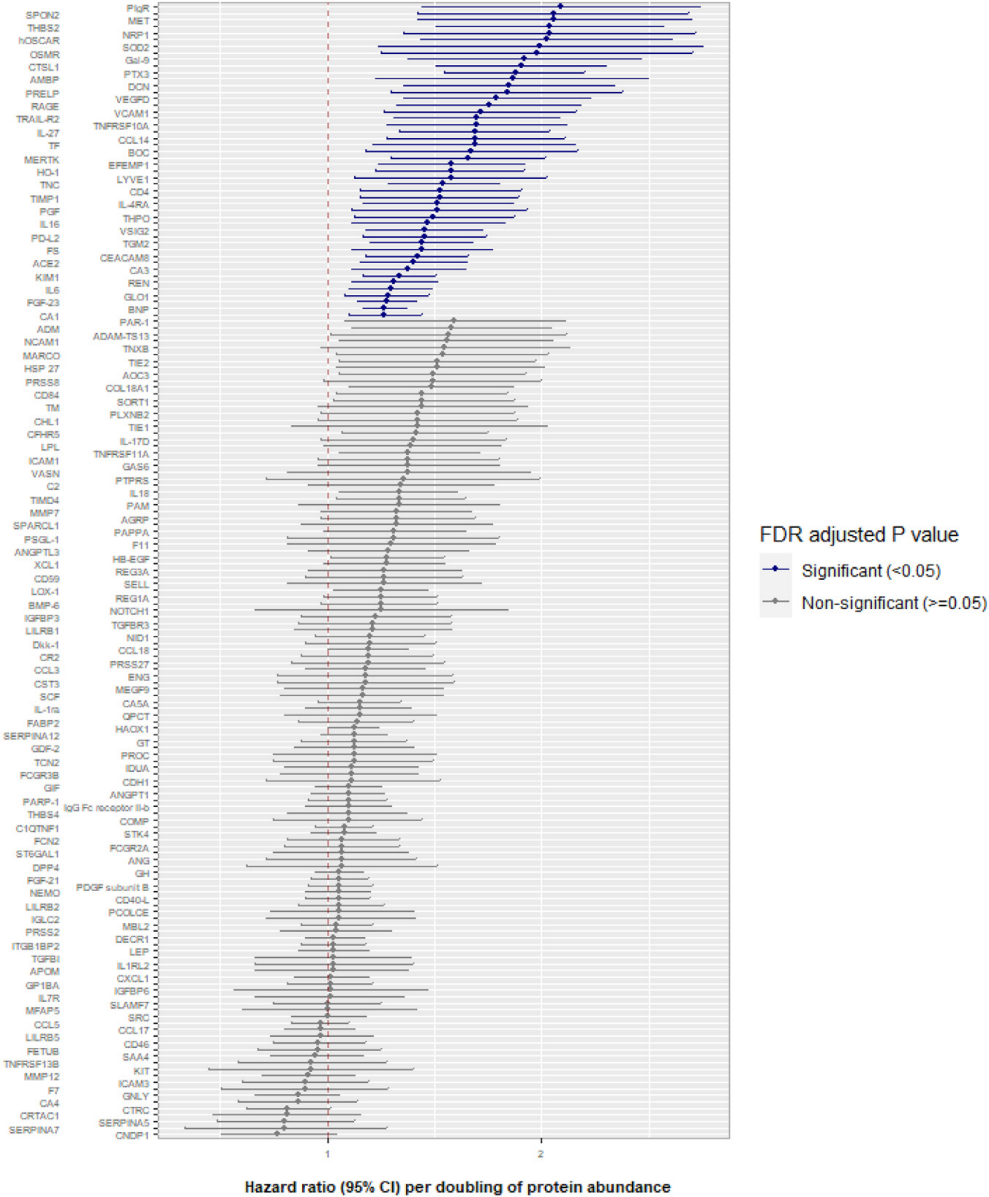
Associations between protein biomarkers and risk of MACE: discovery cohort. Some proteins have confidence intervals that do not cross 1 yet remain nonsignificant (grey), reflecting adjustment for multiple comparisons using the Benjamini and Hochberg false discovery rate method.^14^

Out of the 173 proteins tested in the discovery cohort, 102 proteins reached the FDR-adjusted *P* value (*P*^FDR^) significance threshold in the univariable analysis and 48 of these proteins remained significant in the multivariable model (Fig 1; full results of all analyses are available in Table S1). In the multivariable analysis, the proteins with the strongest evidence of an association with MACE were pentraxin-related protein 3 (PTX3; hazard ratio [HR] = 1.84; 95% confidence interval [CI], 1.46-2.30; *P*^FDR^ = 2.58 × 10^-5^), which is an acute phase protein; brain natriuretic peptide (HR = 1.20; 95% CI, 1.12-1.29; *P*^FDR^ = 8.70 × 10^-5^); and Cathepsin L1 (CTSL1; HR = 1.87; 95% CI, 1.42-2.46; *P*^FDR^ 4.62 × 10^-4^), which has a key role in intracellular protein catabolism. Of the significant proteins, the greatest increase in MACE risk was associated with polymeric immunoglobulin receptor (PlgR; HR = 2.13; 95% CI, 1.36-3.34; *P*^FDR^ 8.87 × 10^-3^), which is a receptor that facilitates transcytosis of immunoglobulins; Spondin 2 (SPON2; HR = 2.08, 95% CI, 1.34-3.21; *P*^FDR^ 8.87 × 10^-3^), which is a cell adhesion protein; and hepatocyte growth factor receptor (HR = 2.08; 95% CI, 1.34-3.24; *P*^FDR^ 9.08 × 10^-3^), which is a multifunctional cytokine that plays a role in angiogenesis, tumorigenesis, and tissue regeneration.

### Multivariable Analyses: Replication Cohort

The 48 proteins that were significant in the discovery cohort multivariable analyses were tested in the replication cohort, and 9 proteins replicated (*P* value threshold of <0.05; Table 3). Of these replicated proteins, 2 proteins (carbonic anhydrase 1 [CA1], carbonic anhydrase 3 [CA3]) had not previously been identified as associated with cardiovascular outcomes in human studies.

**Table 3:** Risk of MACE per Doubling of Protein Abundance: Replicated Proteins

Protein	Discovery Cohort Hazard Ratio (95% CI)	Replication Cohort Hazard Ratio (95% CI)	Replication Cohort *P* Value
Vascular cell adhesion protein 1	1.64 (1.20-2.24)	1.69 (1.05-2.73)	0.03
Interleukin-27	1.61 (1.26-2.05)	1.43 (1.01-2.02)	0.04
Tenascin	1.45 (1.22-1.74)	1.28 (1.02-1.60)	0.03
Placental growth factor	1.43 (1.08-1.91)	1.68 (1.14-2.48)	0.008
Prointerleukin-16	1.38 (1.08-1.77)	1.45 (1.01-2.06)	0.04
V-set & Ig domain-containing protein 2	1.37 (1.13-1.66)	1.42 (1.01-1.99)	0.04
Carbonic anhydrase 3	1.30 (1.08-1.56)	1.50 (1.13-2.00)	0.006
Fibroblast growth factor 23	1.21 (1.1-1.33)	1.21 (1.07-1.35)	0.001
Carbonic anhydrase 1	1.20 (1.07-1.36)	1.19 (1.01-1.40)	0.04

### Sensitivity Analyses: Traditional Risk Factors, CKD-Specific Risk Factors and Level of Proteinuria

**Figure 2:**
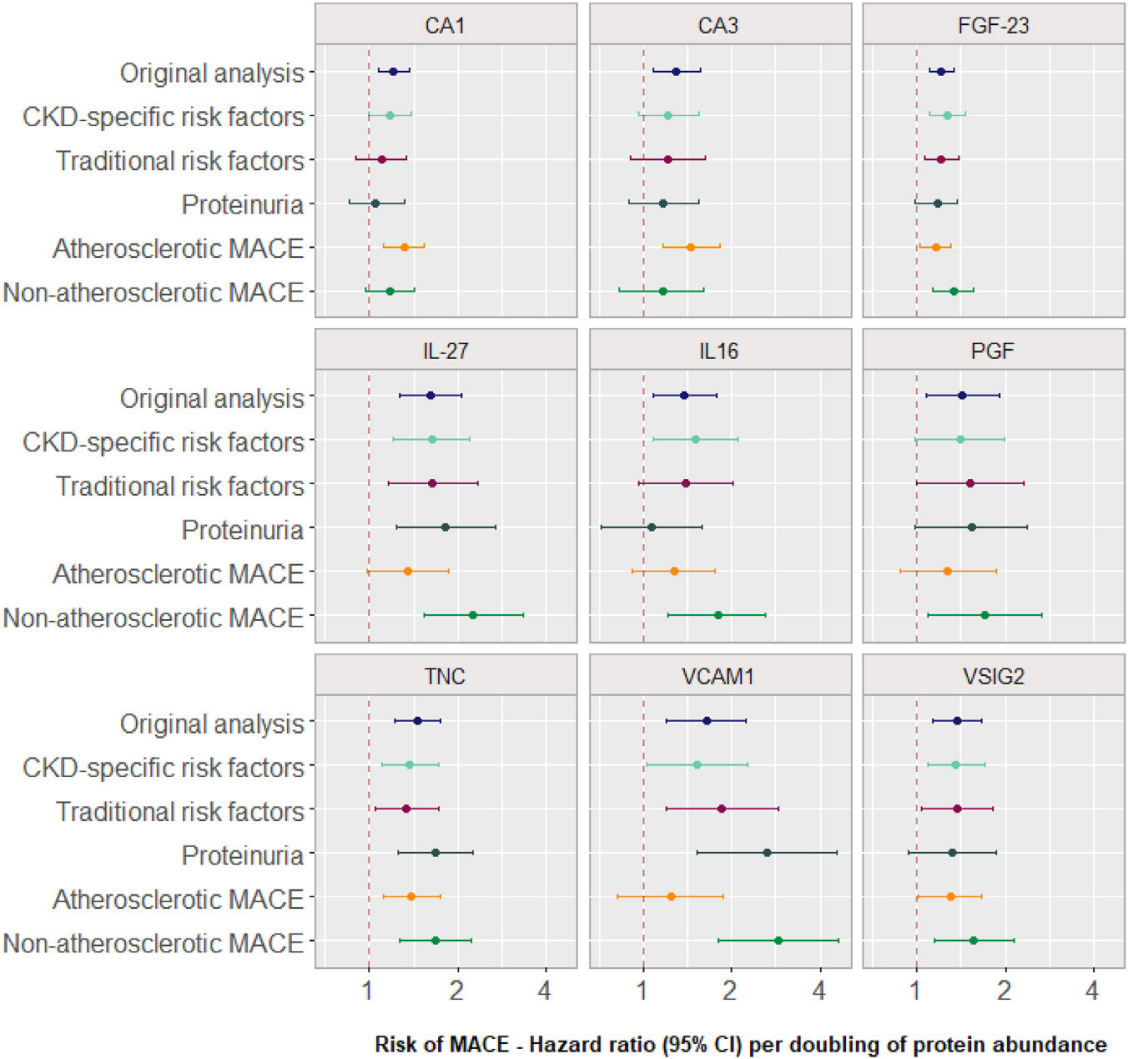
Sensitivity analyses.

After adjustment for traditional risk factors, the relationship between MACE and CA1, CA3, placental growth factor (PGF) and V-set and immunoglobulin domain-containing protein 2 (VSIG2) was mildly attenuated (Fig 2, Table S1). Following adjustment for both traditional and CKD-specific risk factors, the other 6 proteins retained similar effect sizes with MACE but with wider 95% confidence intervals, as expected due to the smaller number of patients in these sensitivity analyses compared with the original analysis (Fig 2, Table S1). After adjustment for the level of proteinuria, only 3 proteins, vascular cell adhesion protein 1 (VCAM1), tenascin (TNC) and interleukin-27 (IL-27), retained a strong association with MACE (*P* < 0.05, Fig 2, Table S1).

### Sensitivity Analyses: Atherosclerotic and Nonatherosclerotic MACE

When the MACE outcome was refined solely to atherosclerotic MACE, CA1, CA3, TNC, FGF-23, and VSIG2 retained a significant association with MACE (*P* < 0.05, Fig 2). In the nonatherosclerotic MACE analyses, CA1 and CA3 were no longer associated with MACE, but the remaining 7 of the 9 proteins maintained their association.

### The 9 Replicated Proteins – Correlation, Functional Annotation, Enrichment Analysis and Protein-Protein Interactions

In our data, PGF and VSIG2 abundance as well as PGF and IL16 levels were moderately correlated (correlation coefficients of 0.62 and 0.58, respectively; Fig S1). The 9 replicated proteins mapped to a variety of molecular functions, biological mechanisms, and protein classes (Fig S2). After taking into account the selection bias from using specific panels of proteins, no protein class was over-represented. Possible interactions between IL16, VCAM1, and PGF as well as CA1 and CA3 were demonstrated in the protein-protein interaction network (Fig S3).

## Discussion

**Figure 3:**
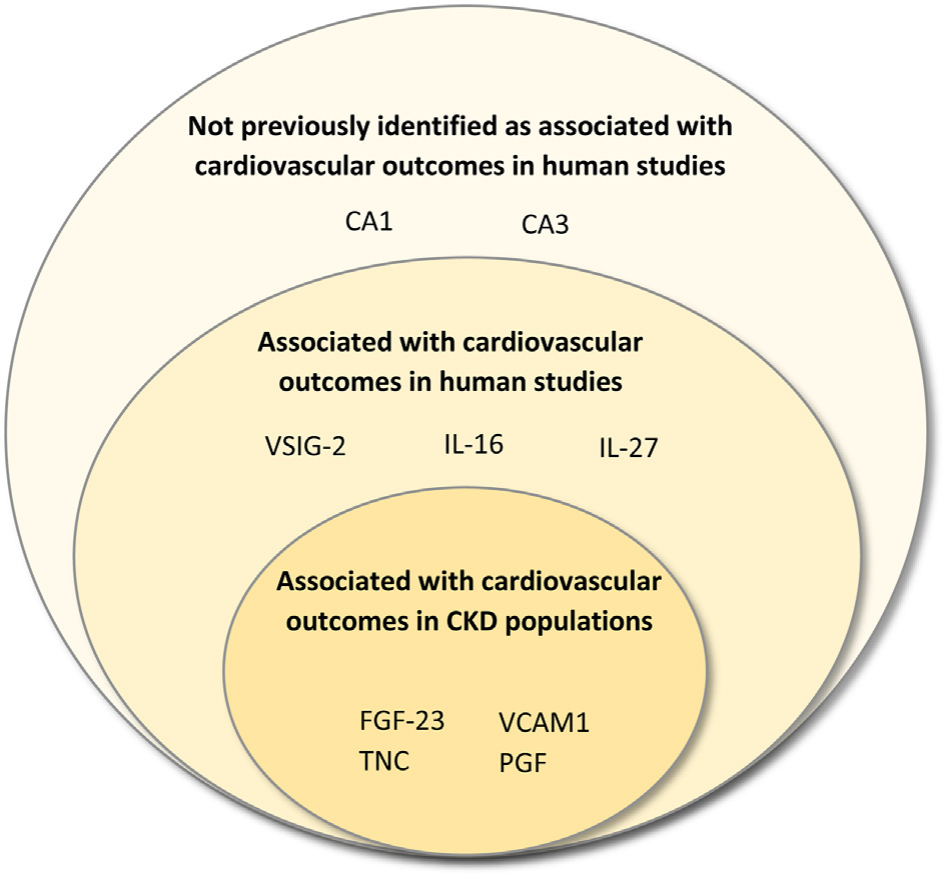
Literature summary of the 9 replicated serum proteins.

We identified associations between proteins and an increased risk of MACE in older people with advanced CKD. Nine proteins were identified in both the discovery and replication cohorts and are proposed as potential biomarkers for MACE amongst an older European CKD population. Our findings corroborate previously reported relationships between FGF-23, VCAM1, PGF, and TNC and cardiovascular outcomes in people with CKD. We also identified relationships between MACE and CA1 and CA3 that have not previously been demonstrated in human studies (Fig 3).

FGF-23 has a key role in phosphate and calcium metabolism and the development of CKD-mineral bone disease. Our findings add further support to the breadth of studies that have identified a relationship between increased FGF-23 and cardiovascular outcomes or mortality, both in the general population and people with CKD.^18^^,^^19^ Interestingly, we found that FGF-23 was associated with MACE, even after adjustment for calcium, phosphate, and parathyroid levels. Despite extensive research interest in FGF-23, debate remains over whether the observed relationship with MACE is likely to be causal or a result of unmeasured confounding factors, possibly reflecting differences in kidney function, secondary hyperparathyroidism, or treatment for renal bone disease.^20^ Indeed, Mendelian randomization studies that used genetic variants associated with FGF-23 as instrumental variables demonstrate a noncausal association between FGF-23 and MACE.^21^^,^^22^ However, whether FGF-23 sits in a causal pathway may not affect its prognostic utility as a MACE biomarker.

Our results contribute to emerging evidence from a small number of studies linking PGF, TNC and VCAM1 with cardiovascular disease in people with CKD. PGF contributes to atherogenesis, vascular inflammation and plaque instability; is associated with mortality and other cardiovascular events; and is not attenuated by age, sex, traditional MACE risk factors or eGFR.^23^ TNC levels independently predict cardiac mortality in dialysis recipients and those with CKD stages G4-5.^24^^,^^25^ Higher VCAM1 levels are associated with elevated all cause and cardiovascular mortality amongst dialysis recipients and negatively correlated with residual kidney function.^26^, ^27^, ^28^ We have also shown the potential for 3 proteins associated with cardiovascular endpoints in the general population to be relevant to those with CKD (V-SIG2, IL-16, and IL-27).^29^, ^30^, ^31^, ^32^ Higher plasma levels of V-SIG2 are associated with incident heart failure.^32^ IL-16 levels have been associated with an unfavorable or cardiovascular risk profile and higher risk of cardiac remodeling and dysfunction.^29^ IL-16 levels help to predict the likelihood of coronary artery events and appear to play a role in the inflammatory and apoptosis pathways underlying incident heart failure.^31^^,^^32^ IL-27 levels are associated with acute MI risk and elevated rates of cardiovascular death, an effect independent of eGFR.^30^ We are the first to our knowledge to report an association between increased serum CA1 and CA3 levels and increased risk of MACE in humans. CA1 and CA3 are isoenzymes that catalyze the reversible hydration of carbon dioxide and are involved in calcification of bone and soft tissue, including changes that occur in pathological states.^33^ Although it has been suggested that these enzymes play a role in the calcification of blood vessels, this has not been conclusively demonstrated.^33^ Overexpression of carbonic anhydrase isoenzymes has also been described in atheromatous plaque.^34^ Given that these proteins were found to be associated with atherosclerotic but not nonatherosclerotic MACE in the sensitivity analysis may support this hypothesis.

Strengths of our study include the replication of our results in a second cohort, the use of hard clinical endpoints as our MACE outcome, and the participants’ similar levels of kidney function. The study of biomarkers is often hampered by a failure to replicate previously reported results. Not only have we replicated our findings, but 4 of the proteins we identified have been shown to be associated with MACE in other studies. As we examined a cohort of older individuals, the use of hard clinical endpoints was particularly important as older individuals may not have sufficient time to accrue the risk associated with surrogate endpoints. All the recruits’ demonstrated similar kidney function at the time of sample collection with an interquartile range of only 7 mL/min/1.73 m^2^; this, alongside adjusting our analysis for eGFR, supports the fact that the differences we have found are not an artefact caused by reduced renal clearance of serum proteins.

There were several limitations in our study. Only a single measurement of the serum proteins was obtained for each person; therefore, we were unable to assess whether within-individual variability of protein levels affect our observed associations with MACE. It is plausible that medication use may have confounded our analyses; for example, in vitro work suggests that many cardiovascular drugs can inhibit CA1.^35^ Also, the number of people included in each of the sensitivity analyses was reduced due to missing data, and CKD-specific risk factors, such as uremic toxins, were not explored. We used a complete case analysis despite missing data for uACR, representing a potential source of bias in our findings. We used directed acyclic graphs to assess the relationship between missing uACR values, confounders, exposure proteins and the MACE outcome. Using the rules of d-separation, we concluded that uACR was likely to be missing not at random. However, it is plausible that missingness is conditional upon other factors, such as kidney function and medication. Imputing missing data using auxiliary variables in the mechanism explaining missingness may have led to different estimates. We did not have sufficient numbers to investigate whether effects were uniform across included subgroups, for example, those with different primary kidney diseases. Finally, our cohort is almost exclusively White European, so our findings may not be generalizable to populations with greater ethnic diversity.

Validation of the findings in independent cohorts is required, and populations with a breadth of age, disease stage, primary kidney disease, and other characteristics should be sought. Potential validation cohorts with protein biomarker data have been identified, but unfortunately there is no overlap between the proteins examined by these cohorts and those which we analyzed.^36^^,^^37^ Therefore, validation would require new funding and data to be generated. If validation work confirms the presence of biomarkers that provide independent prognostic information, then the use of these to guide clinical treatment should be investigated in terms of directing treatment towards those with the greatest potential to benefit and identification of novel therapeutic targets.

To conclude, we identified a shortlist of 9 proteins with potential as MACE biomarkers for older people with advanced CKD. Our findings corroborate previously reported relationships between FGF-23, VCAM1, TNC and PGF and cardiovascular outcomes in people with CKD and highlight 5 other proteins that have not previously been linked with MACE in people with CKD. Furthermore, 3 of these proteins (TNC, VCAM1 andIL-27) have an association with MACE that is independent to known traditional risk factors, CKD-specific risk factors, and level of proteinuria. Further work to validate these proteins in an independent cohort is required.

## Supplementary Material

Supplementary File 1 (PDF) Figures S1-S3; Item S1

Supplementary File 2 (XLS) Table S1.
