# Supplementary material for: Protein Biomarkers and Major Cardiovascular Events in Older People With Advanced CKD: The European Quality (EQUAL) Study
Source: Kidney Med. 2023 Nov 2;6(1):100745. doi: 10.1016/j.xkme.2023.100745 (PMC10757029; doi:10.1016/j.xkme.2023.100745)
Supplement: Supplementary File 1 (PDF) — Figures S1-S3; Item S1 [file mmc1.pdf]

Supplementary Figure S1. Correlation between the 9 replicated proteins

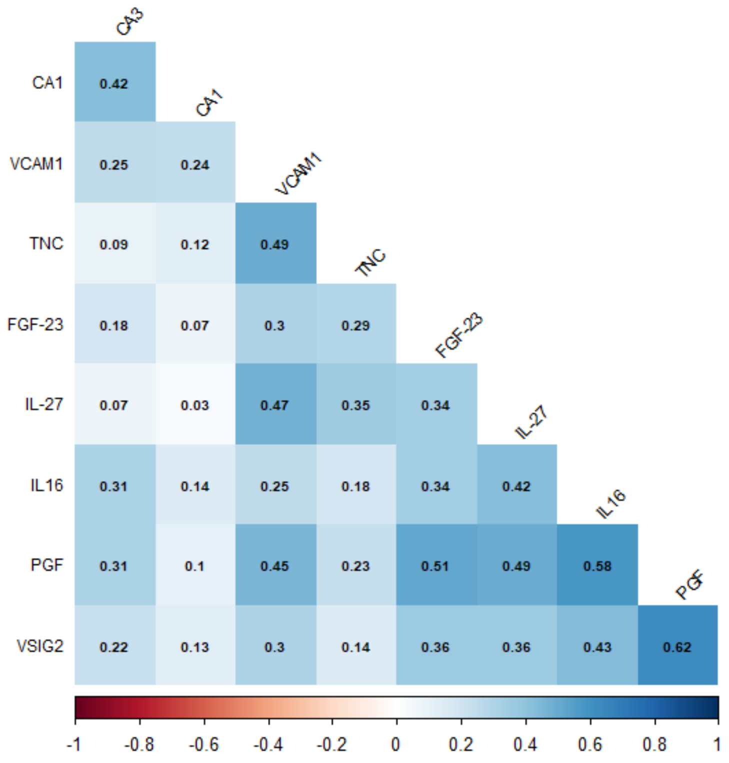

Supplementary Figure S2. Functional annotation - protein classes of the 9 replicated proteins

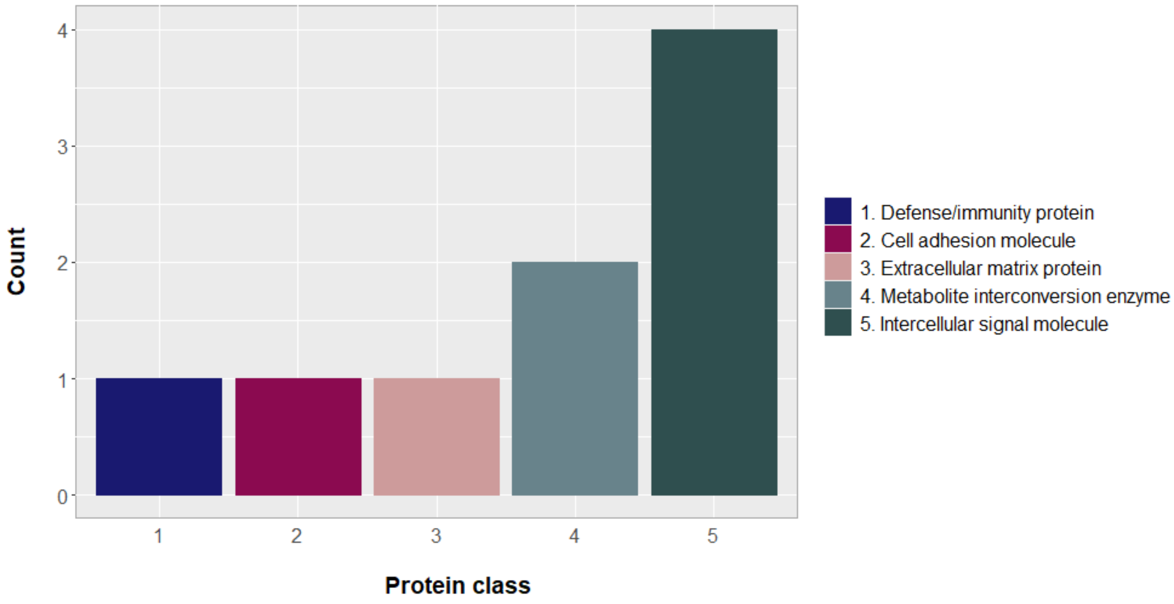

Supplementary Figure S3. Protein-protein interaction network of the 9 replicated proteins

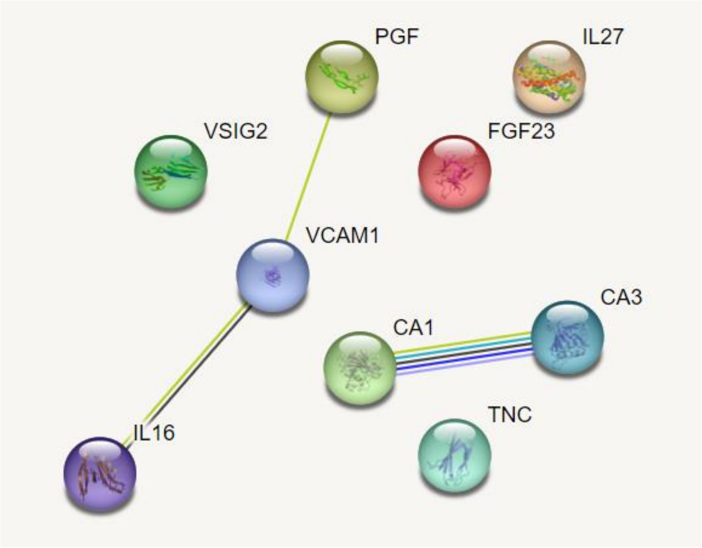

Item S1. List of EQUAL Investigators

NAME	COUNTRY	AFFILIATION
Andreas Schneider	DE	
Anke Torp	DE	
Beate Iwig	DE	
Boris Perras	DE	
Christian Marx	DE	
Christiane Drechsler	DE	
Christof Blaser	DE	
Christoph Wanner	DE	
Claudia Emde	DE	
Detlef Krieter	DE	
Dunja Fuchs	DE	
Ellen Irmler	DE	
Eva Platen	DE	
Hans Schmidt-Gürtler	DE	
Hendrik Schlee	DE	
Holger Naujoks	DE	
Ines Schlee	DE	
Sabine Cäsar	DE	
Joachim Beige	DE	
Jochen Röthele	DE	
Justyna Mazur	DE	
Kai Hahn	DE	
Katja Blouin	DE	
Katrin Neumeier	DE	
Kirsten Anding-Rost	DE	
Lothar Schramm	DE	
Monika Hopf	DE	
Nadja Wuttke	DE	
Nikolaus Frischmuth	DE	
Pawlos Ichtiaris	DE	
Petra Kirste	DE	
Petra Schulz	DE	
Sabine Aign	DE	
Sandra Biribauer	DE	
Sherin Manan	DE	
Silke Röser	DE	
Stefan Heidenreich	DE	
Stephanie Palm	DE	
Susanne Schwedler	DE	
Sylke Delrieux	DE	
Sylvia Renker	DE	
Sylvia Schättel	DE	
Theresa Stephan	DE	
Thomas Schmiedeke	DE	
Thomas Weinreich	DE	
Til Leimbach	DE	
Torsten Stövesand	DE	
Udo Bahner	DE	
Wolfgang Seeger	DE	

Adamasco Cupisti	IT	Unità Operativa Nefrologia e Dialisi 2 Universitaria, Pisa
Adelia Sagliocca	IT	A.O. S.G. Moscati - Avellino
Alberto Ferraro	IT	Nefrologia e Dialisi; Ospedale "S. Giacomo"; Castelfranco
Alessandra Mele	IT	Nefrologia e Dialisi; Ospedale "S. Giacomo"; Castelfranco
Alessandro Naticchia	IT	Policlinico Universitario Agostino Gemelli, Roma
Alex Còsaro	IT	Presidio Ospedaliero di San Bonifacio ULSS 9 Scaligera
Andrea Ranghino	IT	Ospedale S.Luigi , Orbassano (TO)
Andrea Stucchi	IT	UO nefrologia e dialisi, IRCCSS multimedica holding spa, Sesto S.Giovanni, Milano
Angelo Pignataro	IT	Presidio ospedaliero Martini, Torino
Antonella De Blasio	IT	Presidio Ospedaliero "A. Landolfi" Solofra
Antonello Pani	IT	Azienda Ospedaliera Brotzu, Cagliari
Aris Tsalouichos	IT	Ospedale Santa Maria Annunziata - Azienda Usl Toscana
Bellasi Antonio	IT	U.O.C. di Nefrologia, Dialisi, Ospedale S. Anna-Como
Biagio Raffaele Di Iorio	IT	Presidio Ospedaliero "A. Landolfi" Solofra
Butti Alessandra	IT	U.O.C. di Nefrologia, Dialisi, Ospedale S. Anna-Como
Cataldo Abaterusso	IT	Ospedale S. Maria degli Incurabili, Napoli
Chiara Somma	IT	Nefrologia e Dialisi<br>Ospedale "S. Giacomo"<br>Castelfranco Veneto (TV)
Claudia D’alessandro	IT	Azienda Ospedaliera Papa Giovanni XXIII, Bergamo
Claudia Torino	IT	Unità Operativa Nefrologia e Dialisi 2 Universitaria, Pisa
Claudia Zullo	IT	Unità Operativa Nefrologia e Dialisi 2 Universitaria, Pisa
Claudio Pozzi	IT	UO di nefrologia e Dialisi Azienda Ospedaliera Istituti clinici di Perfezionamento Cinisello Balsamo
Daniela Bergamo	IT	SC nefrologia, dialisi Az.Osp CTO Maria Adelaide, Torino
Daniele Ciurlino	IT	UO nefrologia e dialisi,<br>IRCCSS multimedica holding spa,<br>Sesto S.Giovanni, Milano
Daria Motta	IT	Presidio ospedaliero Martini, Torino
Domenico Russo	IT	Azienda Ospedaliera Universitaria Federico II, Napoli
Enrico Favaro	IT	Nefrologia (Ospedale di Castelfranco Veneto)
Federica Vigotti	IT	ASL TO4 Ciriè- Chivasso, Torino, Italia
Ferruccio Ansali	IT	ASL Roma 4, Civitavecchia
Ferruccio Conte	IT	U.O. Nefrologia Dialisi P.O. Cernusco sul Naviglio
Francesca Cianciotta	IT	Azienda Ospedaliero-Universitaria “Consorziale Policlinico” e Università degli Studi “Aldo Moro” di Bari
Francesca Giacchino	IT	Asl TO4 Ivrea
Francesco Cappellaio	IT	ASL Napoli 1 Centro
Francesco Pizzarelli	IT	Ospedale Santa Maria Annunziata - Azienda Usl Toscana
Gaetano Greco	IT	presidio ospedaliero “Nicola Giannettasio” , Rossano
Gaetana Porto	IT	GOM Bianchi Melacrino Morelli Reggio Calabria
Giada Bigatti	IT	Nefrologia e Dialisi IRCCS Multimedica Sesto San Giovanni
Giancarlo Marinangeli	IT	Nefrologia Ospedale di Giulianova
Gianfranca Cabiddu	IT	Azienda Ospedaliera Brotzu, Cagliari
Giordano Fumagalli	IT	Unità Operativa Nefrologia e Dialisi 2 Universitaria, Pisa
Giorgia Caloro	IT	Azienda Ospedaliera- Universitaria Consorziale Policlinico e Università degli Studi “Aldo Moro” di Bari
Giorgina Piccoli	IT	Azienda Ospedaliero-Universitaria San Luigi Gonzaga Orbassano, Torino

Giovanbattista Capasso	IT	Seconda Università di Napoli
Giovanni Gambaro	IT	Policlinico Universitario Agostino Gemelli, Roma
Giuliana Tognarelli	IT	AOU San Luigi Gonzaga, Orbassano, Torino
Giuseppe Bonforte	IT	Azienda Ospedaliera “S. Anna”, Como
Giuseppe Conte	IT	Ospedale S. Maria degli Incurabili, Napoli
Giuseppe Toscano	IT	Ospedale Mazzini, Teramo
Goffredo Del Rosso	IT	Ospedale Mazzini, Teramo
Irene Capizzi	IT	Ospedale S.Luigi , Orbassano (TO)
Ivano Baragetti	IT	UO di nefrologia e Dialisi Azienda Ospedaliera Istituti clinici di Perfezionamento Cinisello Balsamo
Lamberto Oldrizzi	IT	Presidio Ospedaliero di San Bonifacio ULSS 9 Scaligera
Loreto Gesualdo	IT	Università degli studi di Bari Aldo Moro
Luigi Biancone	IT	SC nefrologia, dialisi Az.Osp CTO Maria Adelaide, Torino
Manuela Magnano	IT	Ospedale Mazzini, Teramo
Marco Ricardi	IT	Unità Operativa di Nefrologia e Dialisi, Ospedale "Guglielmo da Saliceto", Piacenza
Maria Di Bari	IT	Nefrologia Ospedale di Giulianova
Maria Laudato	IT	Seconda Università di Napoli
Maria Luisa Sirico	IT	Presidio Ospedaliero "A. Landolfi" Solofra
Martina Ferraresi	IT	Nefrologia, A.S.O.U. San Luigi, Orbassano, Torino
Michele Provenzano	IT	Università degli studi Magna Graecia di Catanzaro
Moreno Malaguti	IT	ASL Roma 4, Civitavecchia
Nicola Palmieri	IT	U.O. Nefrologia Dialisi P.O. Cernusco sul Naviglio
Paola Murrone	IT	Azienda Ospedaliera San Giovanni Addolorata, Roma
Pietro Cirillo	IT	Università degli studi di Bari Aldo Moro
Pietro Dattolo	IT	Ospedale Santa Maria Annunziata - Azienda Usl Toscana
Pina Acampora	IT	Seconda Università di Napoli
Rita Nigro	IT	Università degli Studi di Napoli, Federico II
Roberto Boero	IT	Presidio ospedaliero Martini, Torino
Roberto Scarpioni	IT	Unità Operativa di Nefrologia e Dialisi , Ospedale "Guglielmo da Saliceto", Piacenza
Rosa Sicoli	IT	ASL Roma 4, Civitavecchia
Rosella Malandra	IT	Unità Operativa Complessa di Nefrologia e Dialisi, ASL
Silvana Savoldi	IT	ASL TO4, Ivrea
Silvio Bertoli	IT	UO nefrologia e dialisi, IRCCSS multimedica holding spa, Sesto S.Giovanni, Milano
Silvio Borrelli	IT	A.O. S.G. Moscati - Avellino
Stefania Maxia	IT	Azienda Ospedaliera Brotzu, Cagliari
Stefano Maffei	IT	SC nefrologia, dialisi Az.Osp CTO Maria Adelaide, Torino
Stefano Mangano	IT	U.O.C. di Nefrologia, Dialisi, Ospedale S. Anna-Como
Teresa Cicchetti	IT	presidio ospedaliero “Nicola Giannettasio” , Rossano
Tiziana Rappa	IT	Ospedale S. Maria degli Incurabili, Napoli
Valentina Palazzo	IT	Azienda Ospedaliero-Universitaria “Consorziale Policlinico” e Università degli Studi “Aldo Moro” di Bari
Walter De Simone	IT	A.O. S.G. Moscati - Avellino
Anita Schrander	NL	Alrijne Ziekenhuis, Leiderdorp, The Netherlands
Bastiaan van Dam	NL	Medisch Centrum Alkmaar, Alkmaar, The Netherlands
Carl Siegert	NL	OLVG locatie West, Amsterdam, The Netherlands
Carlo Gaillard	NL	UMCG, Groningen, The Netherlands

Charles Beerenhout	NL	Maxima Medisch Centrum, Veldhoven, The Netherlands
Cornelis Verburgh	NL	Spaarne Gasthuis, Haarlem, The Netherlands
Cynthia Janmaat	NL	LUMC, Leiden, The Netherlands
Ellen Hoogeveen	NL	Jeroen Bosch Ziekenhuis, Den Bosch, The Netherlands
Ewout Hoorn	NL	Erasmus MC, Rotterdam, The Netherlands
Friedo Dekker	NL	LUMC, Leiden, The Netherlands
Johannes Boots	NL	Maasstad Ziekenhuis, Rotterdam, The Netherlands
Henk Boom	NL	Reinier de Graaf Gasthuis, Delft, The Netherlands
Jan-Willem Eijgenraam	NL	Groene Hart Ziekenhuis, Gouda, The Netherlands
Jeroen Kooman	NL	Academic Hospital Maastricht, Maastricht, The
Joris Rotmans	NL	LUMC, Leiden, The Netherlands
Kitty Jager	NL	Amsterdam UMC location AMC, Amsterdam, The
Liffert Vogt	NL	Amsterdam UMC location AMC, Amsterdam, The
Maarten Raasveld	NL	Dijklander Ziekenhuis, Hoorn, The Netherlands
Marc Vervloet	NL	Amsterdam UMC location VUMC, Amsterdam
Marjolijn van Buren	NL	HagaZiekenhuis, The Hague, The Netherlands
Merel van Diepen	NL	LUMC, Leiden, The Netherlands
Nicholas Chesnaye	NL	Amsterdam UMC location AMC, Amsterdam, The
Paul Leurs	NL	ADRZ, Goes, The Netherlands
Pauline Voskamp	NL	LUMC, Leiden, The Netherlands
Peter Blankestijn	NL	UMCU, Utrecht, The Netherlands
Sadie van Esch	NL	Elisabeth-TweeSteden Ziekenhuis, Tilburg, The
Siska Boorsma	NL	Laurentius Ziekenhuis, Roermond, The Netherlands
Stefan Berger	NL	UMCG, Groningen, The Netherlands
Constantijn Konings	NL	Catharina Ziekenhuis, Eindhoven, The Netherlands
Zeynep Aydin	NL	Franciscus Gasthuis, Rotterdam, The Netherlands
Aleksandra Musiała	PL	Department of Nephrology and Transplantation Medicine, Wroclaw Medical University, Wroclaw, Poland
Anna Szymczak	PL	Department of Nephrology and Transplantation Medicine, Wroclaw Medical University, Wroclaw, Poland
Ewelina Olczyk	PL	Department of Nephrology and Transplantation Medicine, Wroclaw Medical University, Wroclaw, Poland
Hanna Augustyniak-Bartosik	PL	Department of Nephrology and Transplantation Medicine, Wroclaw Medical University, Wroclaw, Poland
Ilona Miśkowiec-Wiśniewska	PL	Medicine, The Ludwik Rydygier Collegium Medicum, Nicolaus Copernicus University
Jacek Manitius	PL	Medicine, The Ludwik Rydygier Collegium Medicum, Nicolaus Copernicus University
Joanna Pondel	PL	Department of Nephrology and Transplantation Medicine, Wroclaw Medical University, Wroclaw, Poland
Kamila Jędrzejak	PL	Department of Nephrology and Transplantation Medicine, Wroclaw Medical University, Wroclaw, Poland
Katarzyna Nowańska	PL	Department of Nephrology and Transplantation Medicine, Wroclaw Medical University, Wroclaw, Poland
Łukasz Nowak	PL	Department of Nephrology, Endocrinology and Metabolic Diseases, Medical University of Silesia, Katowice, Poland
Maciej Szymczak	PL	Department of Nephrology and Transplantation Medicine, Wroclaw Medical University, Wroclaw, Poland
Magdalena Durlik	PL	Internal Medicine, Medical University of Warsaw, Warsaw, Poland.

Szyszkowska Dorota	PL	Internal Medicine, Medical University of Warsaw, Warsaw, Poland.
Teresa Nieszporek	PL	Department of Nephrology, Endocrinology and Metabolic Diseases, Medical University of Silesia, Katowice, Poland
Zbigniew Heleniak	PL	Department of Nephrology, Transplantology and Internal Medicine, Medical University of Gdansk, Gdańsk, Poland.
Andreas Jonsson	SE	
Anna-Lena Blom	SE	
Björn Rogland	SE	
Carin Wallquist	SE	
Denes Vargas	SE	
Emöke Dimény	SE	
Fredrik Sundelin	SE	
Fredrik Uhlin	SE	
Gunilla Welander	SE	
Isabel Bascaran Hernandez	SE	
Knut-Christian Gröntoft	SE	
Maria Stendahl	SE	
Maria Svensson	SE	
Marie Evans	SE	
Olof Heimburger	SE	
Pavlos Kashioulis	SE	
Stefan Melander	SE	
Tora Almquist	SE	
Ulrika Jensen	SE	
Alistair Woodman	UK	South Eastern Health and Social Care Trust, Dundonald, Northern Ireland
Anna McKeever	UK	Manchester Royal Infirmary, Manchester, England
Asad Ullah	UK	Royal Liverpool and Broadgreen University Hospital,
Barbara McLaren	UK	NHS Greater Glasgow and Clyde, Glasgow, Scotland
Camille Harron	UK	Northern Health and Social Care Trust, Antrim, Northern
Carla Barrett	UK	Manchester Royal Infirmary, Manchester, England
Charlotte O'Toole	UK	Manchester Royal Infirmary, Manchester, England
Christina Summersgill	UK	Salford Royal NHS Foundation Trust, Salford, England
Colin Geddes	UK	NHS Greater Glasgow and Clyde, Glasgow, Scotland
Deborah Glowski	UK	Altnagelvin Renal Research, Derry, Northern Ireand
Deborah McGlynn	UK	NHS Greater Glasgow and Clyde, Glasgow, Scotland
Dympna Sands	UK	Southern Trust, Newry, Northern Ireland
Fergus Caskey	UK	University of Bristol, Bristol, England & North Bristol NHS Trust, Bristol, England
Geena Roy	UK	Manchester Royal Infirmary, Manchester, England
Gillian Hirst	UK	Manchester Royal Infirmary, Manchester, England
Hayley King	UK	NHS Greater Glasgow and Clyde, Glasgow, Scotland
Helen McNally	UK	North Bristol NHS Trust, Bristol, England
Houda Masri-Senghor	UK	Manchester Royal Infirmary, Manchester, England
Hugh Murtagh	UK	Belfast Health and Social Care Trust, Belfast, Northern
Hugh Rayner	UK	Heart of England NHS Foundation Trust, Birmingham,
Jane Turner	UK	Manchester Royal Infirmary, Manchester, England
Joanne Wilcox	UK	University Hospitals Birmingham, Birmingham, England
Jocelyn Berdeprado	UK	East and North Hertfordshire NHS Trust, Stevenage,

Jonathan Wong	UK	East and North Hertfordshire NHS Trust, Stevenage,
Joyce Banda	UK	Manchester Royal Infirmary, Manchester, England
Kirsteen Jones	UK	NHS Greater Glasgow and Clyde, Glasgow, Scotland
Lesley Haydock	UK	Salford Royal NHS Foundation Trust, Salford, England
Lily Wilkinson	UK	East and North Hertfordshire NHS Trust, Stevenage,
Margaret Carmody	UK	Heart of England NHS Foundation Trust, Birmingham,
Maria Weetman	UK	Oxford University Hospitals NHS Foundation Trust,
Martin Joinson	UK	University Hospitals Birmingham, Birmingham, England
Mary Dutton	UK	University Hospitals Birmingham, Birmingham, England
Michael Matthews	UK	Northern Health and Social Care Trust, Antrim, Northern
Neal Morgan	UK	Southern Trust, Newry, Northern Ireland
Nina Bleakley	UK	Belfast Health and Social Care Trust, Belfast, Northern
Paul Cockwell	UK	University Hospitals Birmingham, Birmingham, England
Paul Roderick	UK	University of Southampton, Southampton, England
Phil Mason	UK	Oxford University Hospitals NHS Foundation Trust,
Philip Kalra	UK	Salford Royal NHS Foundation Trust, Salford, England
Rincy Sajith	UK	Manchester Royal Infirmary, Manchester, England
Sally Chapman	UK	Altnagelvin Renal Research, Derry, Northern Ireand
Santee Navjee	UK	East and North Hertfordshire NHS Trust, Stevenage,
Sarah Crosbie	UK	Oxford University Hospitals NHS Foundation Trust,
Sharon Brown	UK	South Eastern Health and Social Care Trust, Dundonald, Northern Ireland
Sheila Tickle	UK	Royal Liverpool and Broadgreen University Hospital,
Suresh Mathavakkannan	UK	East and North Hertfordshire NHS Trust, Stevenage,
Ying Kuan	UK	Altnagelvin Renal Research, Derry, Northern Ireand
